# Cu_14_ Cluster with Partial Cu(0) Character: Difference in Electronic Structure from Isostructural Silver Analog

**DOI:** 10.1002/advs.201900833

**Published:** 2019-07-26

**Authors:** Yan‐Ling Li, Jie Wang, Peng Luo, Xiao‐Hong Ma, Xi‐Yan Dong, Zhao‐Yang Wang, Chen‐Xia Du, Shuang‐Quan Zang, Thomas C. W. Mak

**Affiliations:** ^1^ College of Chemistry and Molecular Engineering Zhengzhou University Henan 450001 P. R. China; ^2^ College of Chemistry and Chemical Engineering Henan Polytechnic University Jiaozuo 454000 P. R. China; ^3^ Department of Chemistry The Chinese University of Hong Kong Shatin New Territories Hong Kong SAR, P. R. China

**Keywords:** analogs, Cu clusters, Cu(0) character, electronic structure, luminescence

## Abstract

An atom‐precise Cu^0^‐containing copper cluster, Cu_14_(C_2_B_10_H_10_S_2_)_6_(CH_3_CN)_8_ (abbreviated as Cu_14_‐8CH_3_CN) is reported, which is synthesized via a simultaneous reduction strategy and fully characterized by single‐crystal X‐ray diffraction, ESI‐TOF‐MS, and X‐ray photoelectron spectroscopy. Cu_14_‐8CH_3_CN is the only copper cluster that has a virtually identical silver structural analog, i.e., Ag_14_(C_2_B_10_H_10_S_2_)_6_(CH_3_CN)_8_ (hereafter as Ag_14_‐8CH_3_CN). Nevertheless, density functional theory calculations reveal that the electronic structure of Cu_14_‐8CH_3_CN differs significantly from the superatom electronic configuration of Ag_14_‐8CH_3_CN. Moreover, Cu_14_‐8CH_3_CN shows room‐temperature luminescence and good electrocatalytic activities in the ethanol oxidation reaction and detection of H_2_O_2_. This pair of unprecedented analogous molecular nanoscale systems offer an ideal platform to investigate the fundamental differences between copper and silver in terms of catalytic activity and optical properties.

Atomically precise noble metal nanoclusters (NCs) are a relatively new and emerging class of materials linking atoms and nanoparticles that have gained increasing interest due to their aesthetically pleasing molecular structures and potential applications in nanodevices, catalysis, medicine, and imaging.^1^, ^2^ To date, gold and silver NCs of various sizes are well documented, whereas comparable copper NCs remain scarce.^3^ In particular, copper NCs with metallic character are significantly rare probably due to the lower M^I^/M^0^ half‐cell potential of Cu (0.52 V) versus those of Ag (0.80 V) and Au (1.68 V).^4^ To the best of our knowledge, only six such copper NCs, namely [Cu_20_(C≡CPh)_12_(OAc)_6_)],^5^ [Cu_25_H_22_(PPh_3_)_12_]Cl,^6^ [Cu_29_Cl_4_H_22_(Ph_2_phen)_12_]Cl (Ph_2_phen = 4,7‐diphenyl‐1,10‐phenanthroline),^7^ [Cu_43_Al_12_](Cp*)_12_ (Cp* = η^5^‐C_5_Me_5_),^8^ [Cu_53_(CF_3_COO)_10_(C≡C^t^Bu)_20_Cl_2_H_18_]^+^,^9^ and [Cu_13_(S_2_CN^n^Bu_2_)_6_(C≡CR)_4_](PF_6_) (R = C(O)OMe, C_6_H_4_F)^10^ have been reported. Notably, most of them involved H^−^, which is believed to originate from the reductant NaBH_4_ or Ph_2_SiH_2_; the Cu_25_, Cu_29_, and Cu_53_ clusters contain coordinated H^−^, while the Cu_13_ cluster was prepared from Cu hydride cluster precursor. It is well known that the use of excess reducing agents continually hinders the preparation of monodisperse products and makes their isolation more complex. The presence of H^−^ also usually causes problems regarding its precise location and decreases the stability of the NCs. Thus a synthetic method for copper NCs containing Cu^0^ that avoids the use of H^−^ is of vital consideration.

Despite the kinship of the coinage triad, Au, Ag, and Cu exhibit significantly different nanocluster assembly behavior due to their inherent chemical characteristics. Therefore, analogous nanoclusters of these metals are extremely difficult to prepare even under exactly the same synthetic conditions, although isostructural Au, Ag, and Cu coordination complexes are known in other low‐nuclearity systems, such as M_3_(Pz)_3_ (Pz = pyrazole)^11^ and M_4_(SR)_4_.^12^ Very recently, the Bakr group reported [Ag_25_(SR)_18_]^−^, the only silver nanoparticle identically analogous to [Au_25_(SR)_18_]^−^ in terms of the number of metal atoms, ligand count, superatom electronic configuration, and atomic arrangement, which provided the first model nanoparticle platform for understanding the fundamental differences between silver and gold in terms of nobility, catalytic activity, and optical properties.^13^ However, a detailed study of Cu nanoclusters in this regards remains unavailable.

Reducing‐cum‐protecting agents could be desirable in mixed‐valence cluster assembly. 1,2‐dithiolate‐*o*‐carborane has exhibited intense reducing capability and synchronously converted Ag^I^ to Ag^0^ during cluster formation.^14^ Herein we present the one‐pot self‐reduction synthesis and crystal structure of a fcc‐Cu_14_ cluster with Cu^0^ character, Cu_14_(C_2_B_10_H_10_S_2_)_6_(CH_3_CN)_8_ (Cu_14_‐8CH_3_CN), an analog of the previously reported (Ag_14_‐8CH_3_CN).^14^ This cluster is the first copper nanoparticle with an exact silver analog in terms of size, composition, charge and crystal structure, and thus it provides a platform for direct comparison of the properties of copper and silver nanoparticles by both experimental and theoretical methods. Furthermore, the coordinated CH_3_CN molecules on the cluster's surface could be site‐specifically replaced by 4‐dimethylamino‐benzonitrile (DMABN), affording [Cu_14_(C_2_B_10_H_10_S_2_)_6_(DMABN)_8_]·2.5THF (Cu_14_‐8DMABN). This finding further supports the universality of site‐specific surface modification strategy of metal clusters as proposed by us.^14^, ^15^ Besides, Cu_14_‐8CH_3_CN shows good electrocatalytic activities in ethanol oxidation reaction and detection of H_2_O_2_.

The synthesis of Cu_14_‐8CH_3_CN cluster involves the reaction of Cu(CF_3_COO)_2_ with 1,2‐dithiol‐*o*‐carborane in CH_3_CN‐THF (v/v = 1:1) at room temperature (**Figure** **1**; see the Supporting Information for details). Fast self‐reduction of Cu^2+^ to Cu^+^/Cu^0^ can be clearly visualized by color fading of the CH_3_CN solution of Cu(CF_3_COO)_2_ upon dropwise addition of 1,2‐dithiol‐*o*‐carborane in THF (Video S1, Supporting Information), which is rare in one‐step copper cluster synthesis. Block crystals suitable for single‐crystal X‐ray diffraction (SCXRD) analysis were obtained several days later (Figure S1, Supporting Information). To gain an insight into the possible redox reaction during the formation of the cluster, we analyzed the synthetic reaction solution of Cu_14_‐8CH_3_CN by ESI‐TOF‐MS, where a deboronated [(C_2_B_10_H_10_S_2_)(C_2_B_9_H_10_S_2_)]^−^ coupling species with disulfide bonds (Figure S2, Supporting Information) was found in the mass spectrum. Moreover, a byproduct including the disulfide species [Cu(CH_3_CN)_4_][(C_2_B_10_H_10_S_2_)(C_2_B_9_H_10_S_2_)] (abbreviated as Cu‐Disulfide) was fortunately isolated and well‐characterized by single‐crystal X‐ray structure analysis (Figure S3, Supporting Information). The dissociated B atoms from the carborane were probably converted to borates, as evidenced by the ^11^B NMR spectrum of the reaction solution (Figure S4, Supporting Information). These observations indicate that the copper ions could be reduced by the thiol ligands as has been shown in the syntheses of Au and Ag thiolate nanoclusters or nanoparticles,^16^ and/or by the dissociated B atom, and form the resultant Cu(0)‐containing clusters.

**Figure 1:**
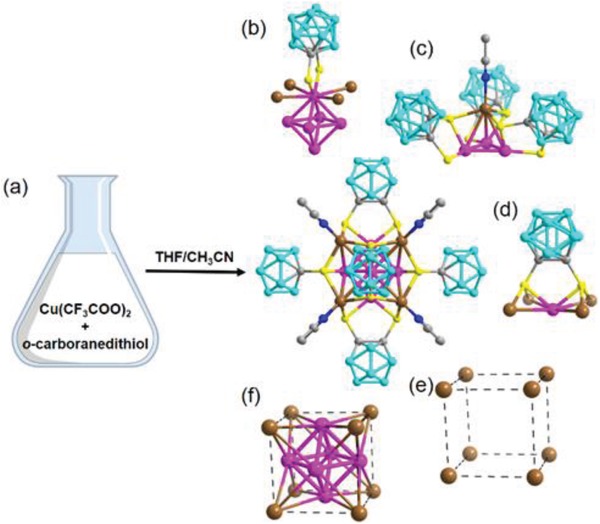
Synthesis and structural dissection of desired Cu_14_‐8CH_3_CN cluster. a) Schematic representation of one‐pot synthesis. b–d) Cu coordination spheres and bonding environment of S atoms. e,f) Cu_6_^4+^ core and Cu_8_^8+^ shell of fcc‐Cu_14_ framework. Color codes: brown and pink = copper; yellow = sulfur; gray = carbon; blue = nitrogen; turquoise = boron.

The as‐synthesized Cu_14_‐8CH_3_CN was characterized by its ^1^H NMR, ^11^B NMR spectra, and energy dispersive X‐ray Spectroscopy (EDS) (Figures S5–S7, Supporting Information). The phase purity of as‐synthesized Cu_14_‐8CH_3_CN was confirmed by in situ powder X‐ray diffraction (PXRD) patterns recorded with milled crystals in the mother liquid (Figure S8, Supporting Information). However, the crystals decomposed very quickly once isolated from the solution, and the PXRD pattern of a dried sample displayed poor crystallinity. However, the retained red emission of the dry sample indicates that the framework of the cluster is robust, and the loss of crystallinity is probably caused by volatility of the lattice or coordinated solvent molecules. This assumption has been confirmed by the recovered PXRD pattern of a long‐term stored (1 month) dry sample soaked in a THF/CH_3_CN mixture (Figure S8, Supporting Information). The stability of Cu_14_‐8CH_3_CN in solution was further studied by monitoring the complex in a CH_2_Cl_2_/CH_3_OH (v/v = 1:1) solution with UV–vis spectroscopy at room temperature. The UV–vis spectra remained essentially unchanged over 24 h, indicating its high stability (**Figure** **2**b). High‐resolution ESI‐TOF‐MS was also conducted to confirm the chemical formula and verify the stability. The excellent match of the experimental and simulated isotope patterns illustrated that the peaks at ≈2169.87, 2210.89, and 2251.91 Da correspond to the [M‐7CH_3_CN+H]^+^, [M‐6CH_3_CN+H]^+^, and [M‐5CH_3_CN+H]^+^ species, respectively, which also indicates easy dissociation of the coordinated CH_3_CN molecules (Figure 2a). The exposed Cu atoms generated by surface coordination bond cleavage might render this compound catalytically active in some reactions. The Cu Auger lines at 1848.55 and 1850.9 eV, obtained from the X‐ray photoelectron spectroscopy (XPS) spectrum as the sum of the Cu 2P_3/2_ binding energy and the LMM Auger kinetic energy, indicate the coexistence of Cu(I) and Cu(0) (Figure S9, Supporting Information).^6^

**Figure 2:**
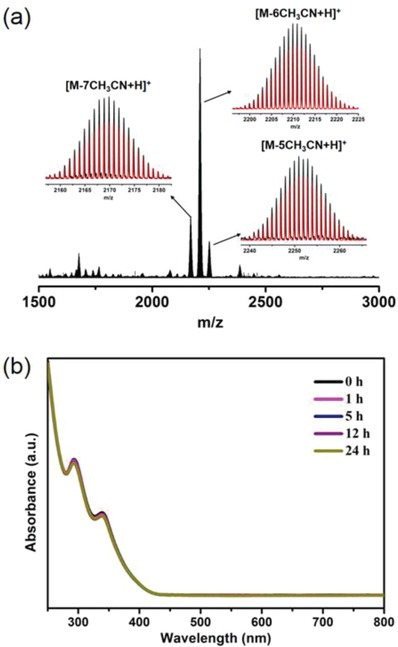
a) Positive mode ESI‐TOF‐MS spectrum of Cu_14_‐8CH_3_CN. Inset: Enlarged portion of the spectrum showing the measured (black) and simulated (red) isotopic distribution patterns (M = Cu_14_(C_2_B_10_H_10_S_2_)_6_(CH_3_CN)_8_). b) Time‐dependent UV–vis spectra of Cu_14_‐8CH_3_CN in CH_2_Cl_2_/CH_3_OH solution (v/v = 1:1, 2.5 × 10^−5^ mol L^−1^).

SCXRD analysis revealed that Cu_14_‐8CH_3_CN crystallizes in cubic space group *Fmm* (Table S1, Supporting Information), being unlike Ag_14_‐8CH_3_CN that crystallizes in triclinic space group *P*.^14^ Cu_14_‐8CH_3_CN contains a discrete cubic Cu_14_ core with six face‐capping bidentate 1,2‐dithiolate‐*o*‐carborane ligands, and eight vertex‐capping CH_3_CN ligands (Figure 1). The structure is identical to that of the Ag_14_‐8CH_3_CN homolog, but the fcc metal framework is more compact (the edge of the cube is 4.098 Å in Cu_14_‐8CH_3_CN, and average 4.458 Å in Ag_14_‐8CH_3_CN) (Figure 1e,f). Each Cu atom in the Cu_6_^4+^ core adopts ten‐coordinate geometry, bonding to four adjacent Cu atoms at the octahedral vertexes with a Cu⋅⋅⋅Cu distance of 2.4926 Å, four Cu atoms from the corresponding face of the cubic Cu_8_^8+^ shell (Cu⋅⋅⋅Cu separation of 2.912 Å), and two S atoms from one 1,2‐dithiolate‐*o*‐carborane ligand (Figure 1b). In the cubic Cu_8_^8+^ shell, each Cu atom adopts a seven‐coordinate geometry, bonding to three S atoms from three different 1,2‐dithiolate‐*o*‐carborane ligands, three Cu atoms of the Cu_6_^4+^ unit, and one CH_3_CN molecule located at a cubic corner (Figure 1c). Each thiolate group acts as a µ_3_‐bridge between one Cu atom from the Cu_6_^4+^ core and two Cu atoms from the Cu_8_^8+^ shell (Figure 1d), with Cu—S bond distances of 2.2564 and 2.367 Å.

The jelliumatic electron count of Cu_14_‐8CH_3_CN is 2 (*n* = 14 − 6 × 2), being the same with that of Ag_14_‐8CH_3_CN. To gain insight into the electronic structure of Cu_14_‐8CH_3_CN and compare it to that of its Ag_14_‐8CH_3_CN analog, density functional theory (DFT) calculations were carried out. The degenerate lowest unoccupied molecular orbitals (LUMO to LUMO+2) indicate strong superatomic P character over the entire Cu_14_ cube, which is highly similar to that of Ag_14_‐8CH_3_CN; however, unlike the S‐symmetric HOMO state of Ag_14_‐8CH_3_CN, the jellium 1S orbital of Cu_14_‐8CH_3_CN shifted down as a low‐lying HOMO‐6 state over the Cu_6_^4+^ kernel (**Figure** **3** and Figure S10, Supporting Information). These findings indicate that the silver and copper cluster analogs with identical composition, molecular structure, and valence electron count do not necessarily have the same electronic structures.

**Figure 3:**
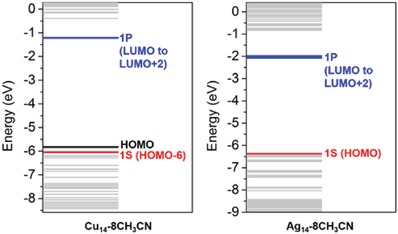
Locations of the superatomic states for Cu_14_‐8CH_3_CN and Ag_14_‐8CH_3_CN. Ag_14_‐8CH_3_CN has the character of the 1S‐symmetric superatomic state in the HOMO level and the 1P‐symmetric state in the LUMO to LUMO+2 levels. Cu_14_‐8CH_3_CN has the character of the 1S‐symmetric superatomic state in the HOMO‐6 level and the 1P‐symmetric state in its LUMO to LUMO+2 level.

We simulated the optical absorption of Cu_14_‐8CH_3_CN with time‐dependent density functional theory (TD‐DFT) to identify the origin of the optical transitions. Compared to the experimental molecular‐like absorption spectrum of a CH_2_Cl_2_‐CH_3_OH solution of dry Cu_14_‐8CH_3_CN crystals, the simulated spectrum displays an analogous profile but had a significant blueshift (**Figure** **4**a). As mentioned above, the coordinated CH_3_CN molecules could dissociate readily. The dry sample probably lost part or all the coordinated CH_3_CN molecules before being used for the UV–vis absorption test, which might account for the observed shift. To prove this, one drop of CH_3_CN was added to the test solution, and the resulting electronic absorption showed an obvious blueshift just as surmised (Figure S11, Supporting Information). Therefore, we simulated the optical absorption of a Cu_14_ model with all CH_3_CN molecules omitted (Figure S12, Supporting Information). In contrast, the calculated spectrum exhibits a dramatic redshift with respect to the experimental spectrum, being accompanied by a slight difference in profile. To devise a compromise, we created a Cu_14_‐4CH_3_CN model bearing only four CH_3_CN ligand molecules (Figure S13, Supporting Information). The simulated optical absorption spectrum matches the experimental spectrum much better, with a major band at 287 nm (HOMO‐20 to LUMO) and a weaker band at 323 nm (HOMO‐6 to LUMO+1) corresponding to the experimental peaks at 294 and 339 nm, respectively. The acceptable deviations might be due to the inaccurate CH_3_CN number and positions. A comparison of the absorption spectra of Cu_14_‐8CH_3_CN and its Ag analog Ag_14_‐8py^14^ was shown in Figure S14 (Supporting Information), as Ag_14_‐8CH_3_CN exhibits compromised stability. The obvious difference, which is in accordance to their electronic structures, is attributed to the different nature of the metals.

**Figure 4:**
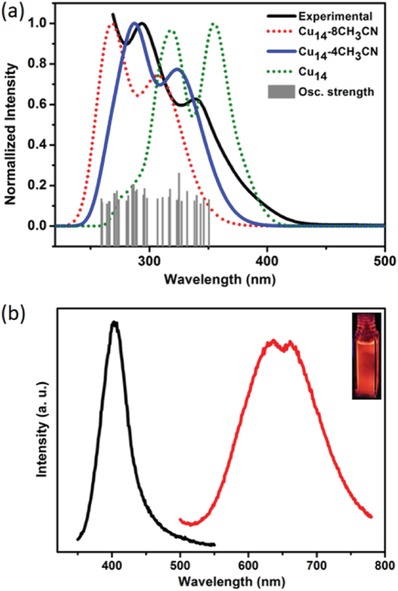
a) Normalized computed optical absorption spectra of Cu_14_‐8CH_3_CN (red dot line), Cu_14_‐4CH_3_CN (blue solid line) and Cu_14_ (green dot line) compared to the experimental data of Cu_14_‐8CH_3_CN (black solid line). Gray bars show the individual transitions (delta‐function‐like peaks showing the relative oscillator strengths) (Table S2, Supporting Information). The continuous computational spectra are sums of Gaussian smoothed individual transitions (width of 15 nm). b) Excitation (black trace) and emission (red trace) spectra of Cu_14_‐8CH_3_CN. Inset: photograph of Cu_14_‐8CH_3_CN in CH_2_Cl_2_ irradiated with 365 nm UV light at room temperature.

Although Cu complexes have been investigated as luminescent materials for decades,[qv: 1h] the reported Cu NCs with Cu(0) character are all nonemissive. Interestingly, Cu_14_‐8CH_3_CN emits bright red light upon UV irradiation in both solid and solution states at room temperature. The luminescence spectrum (λ_ex_ = 400 nm) displays a weakly structured broad emission band with two peaks at 637 and 661 nm (Figure 4b), the profile of which is akin to that of Ag_14_‐8CH_3_CN. The quantum yield of the emission at room temperature is 0.31. The microsecond‐scale emissive lifetime (τ_298 K_ = 5.13 µs) indicates spin‐forbidden triplet phosphorescence. Based on DFT and TDDFT calculations (Figure 4a and Figure S10, Supporting Information), the emission of Cu_14_‐8CH_3_CN might mainly originate from the excited state that arose from S‐type HOMO‐6 and the ligand‐based HOMOs to P‐type LUMOs transitions. Upon photoexcitation, the excited electrons into superatomic 1P orbitals would lead to some distortions of excited states, which might be related to the large Stokes shift and the hump‐like peaks in emission spectra.^17^

The surface modification with pyridyl ligands used for Ag_14_‐8CH_3_CN were not applicable to Cu_14_‐8CH_3_CN, highlighting the difference of silver and copper cluster analogs. To address this problem, 4‐(dimethylamino)benzonitrile was used to replace the CH_3_CN molecules, and eight DMABN molecules occupied all fcc corners as expected, giving surface‐modified Cu_14_‐8DMABN (Figure S15, Supporting Information). The product was characterized by ESI‐MS, ^1^H and ^11^B NMR spectroscopy (Figures S16–S18, Supporting Information). The UV–vis absorption spectrum of Cu_14_‐8DMABN in DMF shows a similar profile to that of Cu_14_‐8CH_3_CN with two bands at 295 and 345 nm, which indicates that surface modification seldom affects the electronic structure of the cluster (Figure S19, Supporting Information). The enhanced absorption at around 295 nm is probably due to overlap of the *n* → π* or π → π* transitions of the DMABN ligands. These achievements further demonstrate that site‐specific replacement of the coordinated solvent molecules in the ligand shell to modify a metal cluster while retaining its metal‐core integrity is feasible and universal.

Development of low‐cost electrocatalysts capable of oxidizing ethanol with high efficiency holds great promise for resolving the impediments to developing practical direct ethanol fuel cells.^18^ The electro‐oxidation reaction of ethanol for Cu_14_‐8CH_3_CN was evaluated in a solution containing 0.1 m KOH and different concentrations of ethanol (Figures S20 and S21, Supporting Information). An oxidative peak at +0.84 V in the forward scan appeared in the presence of 0.5 m C_2_H_5_OH. And peak current rose and peak potential shifts to higher positive position with the increase of the ethanol concentration, verifying an excellent electrocatalytic activity of Cu_14_‐8CH_3_CN in this reaction. The linear relationship between anodic current density and ethanol concentration confirms that the ethanol oxidation process is controlled by the kinetic diffusion of ethanol. PXRD pattern of the sample collected after electrocatalytic reaction well reproduced the simulated one (Figure S22, Supporting Information), indicating that Cu_14_‐8CH_3_CN cluster remains intact.

Cu_14_‐8CH_3_CN clusters were also investigated for electrochemical detection of H_2_O_2_. The CVs of Cu_14_‐8CH_3_CN/GC electrode in 0.1 m phosphate buffer solution (PBS) with different concentrations of H_2_O_2_, which was bubbled with ultrahigh purity nitrogen for at least 15 min prior to the electrochemical measurements, shows that obvious reduction current appears after the introduction of H_2_O_2_ and the reduction current increases with the increase of H_2_O_2_ concentrations (**Figure** **5**a). These findings indicate the high electrochemical activity of Cu_14_‐8CH_3_CN for H_2_O_2_ detection. The amperometric response of Cu_14_‐8CH_3_CN to the successive addition of H_2_O_2_ was investigated at −0.45 V. From the obtained *i*–*t* curve shown in Figure 5b, it can be clearly seen that the reduction current increases with successive addition of H_2_O_2_ and the current can reach a steady state rapidly, indicating the sensitive and fast response of Cu_14_‐8CH_3_CN to the concentration change of H_2_O_2_. The responding current and concentration of H_2_O_2_ display linear relationship (Figure 5a). The limit of detection (LOD) was estimated to be 2.3 × 10^−6^ m based on the three times the standard deviation for the average measurement of blank sample (LOD = 3σs^−1^).

**Figure 5:**
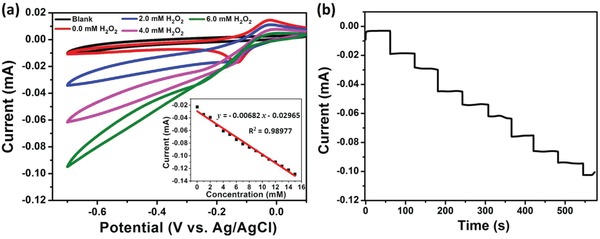
a) CVs of blank electrode (6 × 10^−3^ m H_2_O_2_) and Cu_14_‐8CH_3_CN in 0.1 m PBS with the absence and presence of H_2_O_2_ at different concentrations of 2, 4, and 6 × 10^−3^ m, scan rate: 100 mV s^−1^. Inset: The linear relationship between responding current and concentration of H_2_O_2_. b) The amperometric *i*–*t* curve of Cu_14_‐8CH_3_CN upon successive addition of H_2_O_2_ recorded at −0.45 V.

In summary, we have synthesized and fully characterized Cu_14_(C_2_B_10_H_10_S_2_)_6_(CH_3_CN)_8_ (Cu_14_‐8CH_3_CN), the first copper analog of known Ag_14_‐8CH_3_CN. The similarities and differences in electronic structure and optical property between these Group 11 cluster analogs have been thoroughly investigated by experimental and theoretical methods. In addition, the catalytic activities of Cu_14_‐8CH_3_CN in ethanol electro‐oxidation reaction and electrochemical detection of H_2_O_2_ were evaluated. This work provides a perspective for preparing Cu^0^‐containing clusters via a self‐reduction procedure, and it serves as a model platform for direct comparison of the physicochemical properties of copper and silver at the nanoscale level.

[CCDC 1861371, 1861372, 1886055 contains the supplementary crystallographic data for this paper. These data can be obtained free of charge from The Cambridge Crystallographic Data Centre via www.ccdc.cam.ac.uk/data_request/cif].

## Conflict of Interest

The authors declare no conflict of interest.

## Supporting information

Supplementary

Supplementary
