# Supplementary material for: Cu14 Cluster with Partial Cu(0) Character: Difference in Electronic Structure from Isostructural Silver Analog
Source: Adv Sci (Weinh). 2019 Jul 26;6(18):1900833. doi: 10.1002/advs.201900833 (PMC6755520; doi:10.1002/advs.201900833)
Supplement: Supplementary file 1 — Supplementary [file ADVS-6-1900833-s002.pdf]

Copyright WILEY-VCH Verlag GmbH & Co. KGaA, 69469 Weinheim, Germany, 2019.

ADVANCED SCIENCE
Open Access

Supporting Information

for *Adv. Sci.,* DOI: 10.1002/advs.201900833

Cu_{14} Cluster with Partial Cu(0) Character: Difference in Electronic Structure from Isostructural Silver Analog

Yan-Ling Li, Jie Wang, Peng Luo, Xiao-Hong Ma, Xi-Yan Dong, Zhao-Yang Wang, Chen-Xia Du, Shuang-Quan Zang,* and Thomas C. W. Mak*

WILEY-VCH

Copyright WILEY-VCH Verlag GmbH & Co. KGaA, 69469 Weinheim, Germany, 2019.

Supporting Information

Cu_{14} Cluster with Partial Cu(0) Character : Difference in Electronic Structure from Isostructural Silver Analog

Yan-Ling Li, Jie Wang, Peng Luo, Xiao-Hong Ma, Xi-Yan Dong, Zhao-Yang Wang, Chen-Xia Du, Shuang-Quan Zang,* and Thomas C. W. Mak*

1. Experimental

1.1 Reagents. 1,2-dithiol-*o*-carborane were prepared by a literature method.[1] All other reagents and solvents used were of commercially available reagent grade and were used without any additional purification.

1.2 Apparatus and Characterization

UV-vis absorption spectra were recorded with a U-2000 spectrophotometer. The HRESI-TOF-MS spectrum of **Cu_{14}-8CH_3CN** was collected on a SolariX 9.4T ICR spectrometer. The HRESI-TOF-MS spectra of **Cu_{14}-8DMABN** and the reaction solution were collected on an AB Sciex X500R Q-TOF spectrometer. ^{1}H and ^{11}B NMR spectra were recorded using Bruker AV300 spectrometer. Chemical shifts are expressed in parts per million (ppm) downfield from internal TMS. EDS measurement of **Cu_{14}-8CH_3CN** was carried out using Zeiss Sigma 500 system. X-ray photoelectron spectroscopy (XPS) measurements were performed with a VG Scientific ESCALAB 250 instrument equipped with a monochromatic Al Kα x-ray source (hν = 1486.8 eV). Samples were loaded into a custom built air-free sample holder, under a N_2 atmosphere. Prior to data collection, a baseline vacuum of 1.07 x 10^{-9} mbar was achieved. For high resolution scans, a band pass energy of 20 eV was used. Binding energies were calibrated using the C 1s peak of adventitious carbon at 284.8 eV. The peak positions in the Cu 2p region and the LMM Auger emission were determined using the Casa XPS software package.

Powder X-ray diffraction (PXRD). PXRD data were collected at room temperature in air using an X' Pert PRO diffractometer (Cu Kα, λ = 1.54178 Å). In situ PXRD patterns were collected on samples immersed in the mother liquor on a Rigaku XtaLAB Pro diffractometer with Cu-Kα radiation.

Luminescence measurements. Luminescence spectra were recorded on a HORIBA FluoroLog-3 fluorescence spectrometer. Luminescence decay was measured on a HORIBA Scientific Fluorolog-3 spectrofluorometer equipped with a 355 nm laser operating in time-correlated single photon counting mode (TCSPC) with a resolution time of 680 µs. The photoluminescent quantum efficiency in solution (1.6×10^{-3} mol/L) was measured using an integrating sphere on a HORIBA Scientific Fluorolog-3 spectrofluorometer.

Single-crystal X-ray diffraction analysis (SCXRD). SCXRD measurements were performed on a Rigaku XtaLAB Pro diffractometer with Cu-Kα radiation (λ = 1.54184 Å) at 200 K for **Cu_{14}-8CH_3CN** and 150 K for **Cu_{14}-8DMABN**. Data collection and reduction were performed

using the program CrysAlisPro.[2] The intensities were corrected for absorption using an empirical method implemented in the SCALE3 ABSPACK scaling algorithm. The structures were solved with intrinsic phasing methods (*SHELXT-2015*)[3] for **Cu_{14}-8DMABN** and direct methods (*SHELXS-2015*)[4] for **Cu_{14}-8CH_3CN** and were refined by full-matrix least squares on F^2 using *OLEX2*,[5] which utilizes the *SHELXL-2015* module.[3] All non-hydrogen atoms were refined with anisotropic thermal parameters, and the hydrogen atoms were included at idealized positions. In **Cu_{14}-8CH_3CN**, the high symmetry, symmetry inconsistency (symmetry of *Fm*-3*m* space group is higher than that of the carborane moiety) and the site disorder of the 1,2-dithiolate-*o*-carborane made it difficult to solve the carborane structure in the correct icosahedron model. Fortunately, we succeeded in distinguishing two sets of half-occupied 1,2-dithiolate-*o*-carborane as icosahedral carborane moieties. To tackle the connectivity problems for theoretical H addition to B atoms, a few "free" commands were used. There was a large solvent-accessible void volume in the crystals of **Cu_{14}-8CH_3CN**, which was occupied by highly disordered solvent molecules. No satisfactory disorder model could be found; therefore, the *Solvent Mask* program implemented in *OLEX2*[5] was used to remove background residual electron densities. In **Cu-Disulfide**, one B atom in the carborane was refined with 0.5 occupancy to realize the coupling species with one *closo* carborane and one deboronated carborane. The B-H-B bridging H atom could not be localized based on Difference Fourier maps. The crystal structures are visualized using DIAMOND 3.2.[6]

1.3 Density functional theory (DFT) calculations. DFT and time-dependent density functional theory (TD-DFT) calculations were performed with Gaussian 16[7] under the Perdew−Burke−Ernzerhof (PBE) functional.[8] All calculations were conducted using the Def2-SVP basis set for all atoms.[9] The single-crystal structure was chosen as the initial guess for ground-state optimization, and all reported stationary points were verified as true minima by the absence of negative eigenvalues in the vibrational frequency analysis. The calculated absorption spectra were obtained from GaussSum 2.1.[10] Hirshfeld population analysis was conducted by Multiwfn 3.4.[11]

1.4 Synthesis

Preparation of Cu_{14}-8CH_3CN. A solution of 1,2-dithiol-*o*-carborane (10.4 mg, 0.05 mmol) in 2 mL of THF was added dropwise to an acetonitrile (2 mL) solution of $Cu(CF_3COO)_2$ (14.5 mg, 0.05 mmol) under stirring. The resultant light yellow solution was evaporated slowly at room temperature for 3 days to afford light green block crystals suitable for SCXRD analysis. Yield: 1.1 mg, 12% based on Cu.

Preparation of Cu_{14}-8DMABN. 1,2-dithiol-*o*-carborane (10.4 mg, 0.05 mmol) in 2 mL of THF was mixed with $Cu(CF_3COO)_2$ (14.5 mg, 0.05 mmol) in 2 mL of CH_3OH, followed by the addition of DMABN (13.0 mg, 0.1 mmol) in 0.5 mL of THF. The resultant solution was kept at room temperature for several days to afford prismatic crystals. Yield: 5.3 mg, 45% based on Cu.

2. Selected spectra and data referred in the paper

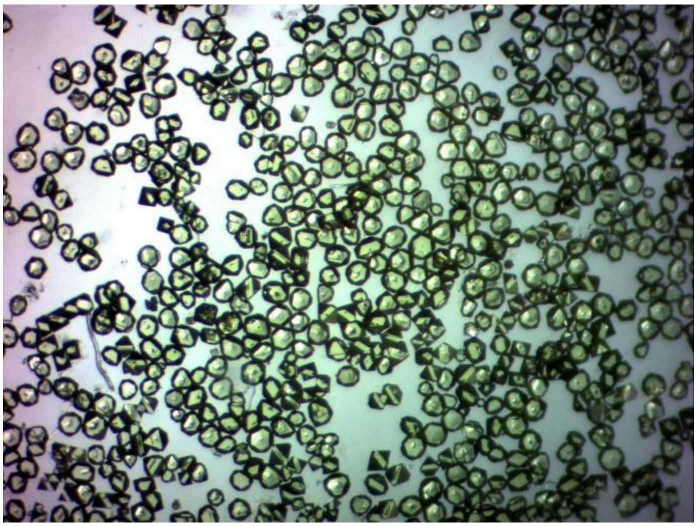

Figure S1. Image of single crystals of **Cu_{14}-8CH_3CN**.

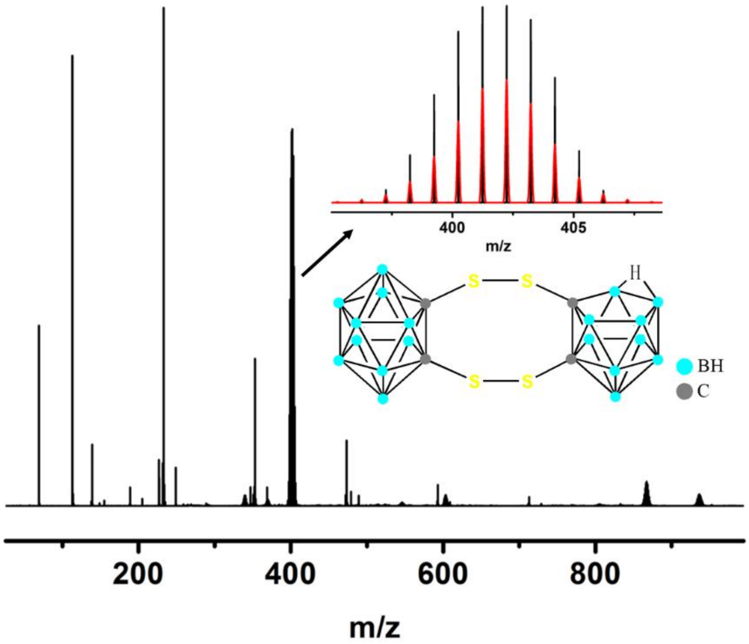

Figure S2. Negative-mode ESI-TOF-MS spectrum recorded with the synthetic reaction solution of **Cu_{14}-8CH_3CN**. Inset: Enlarged portion of the spectrum showing the measured (black) and simulated (red) isotopic distribution patterns, and the structure of the corresponding $[(C_2B_{10}H_{10}S_2)\ (C_2B_9H_{10}S_2)]^-$ species. m/z calcd for $[(C_2B_{10}H_{10}S_2)(C_2B_9H_{10}S_2)]^-$: 402.2335. Found: 402.2349.

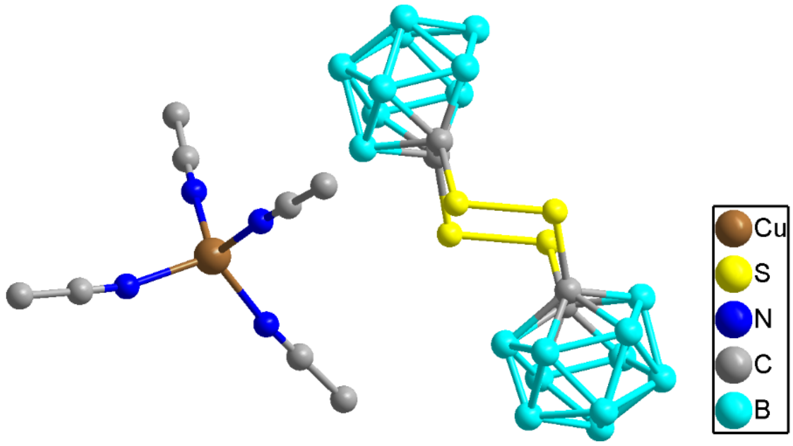

Figure S3. Molecular ions in the crystal structure of **Cu-Disulfide**. The B-H-B bridging H atom could not be localized, and other H atoms are omitted for clarity.

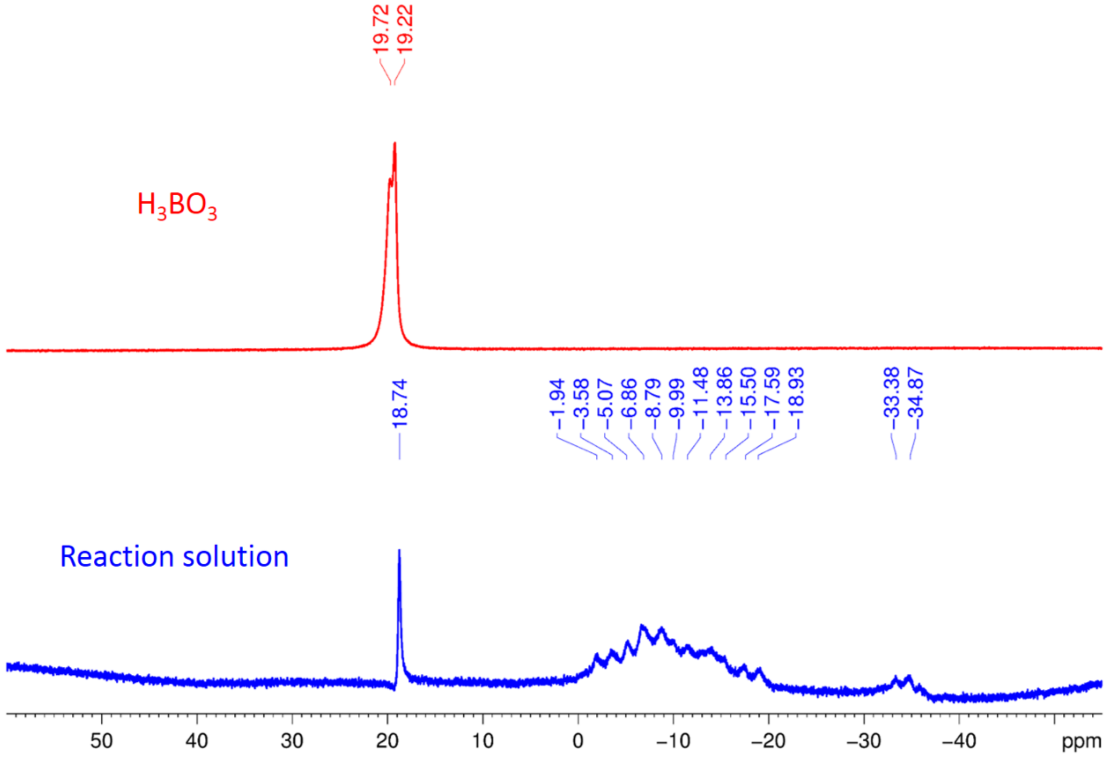

Figure S4. ^{11}B NMR spectrum of H_3BO_3 and the synthetic reaction solution of **Cu_{14}-8CH_3CN**. The sharp peak at 18.74 ppm is in good accordance to the signal of H_3BO_3; the signal from -1.94 to -18.93 ppm is assigned to the *closo*-carborane groups; the weak multiple signal around -34 ppm is tentatively attributed to the deboronated carborane units.[12]

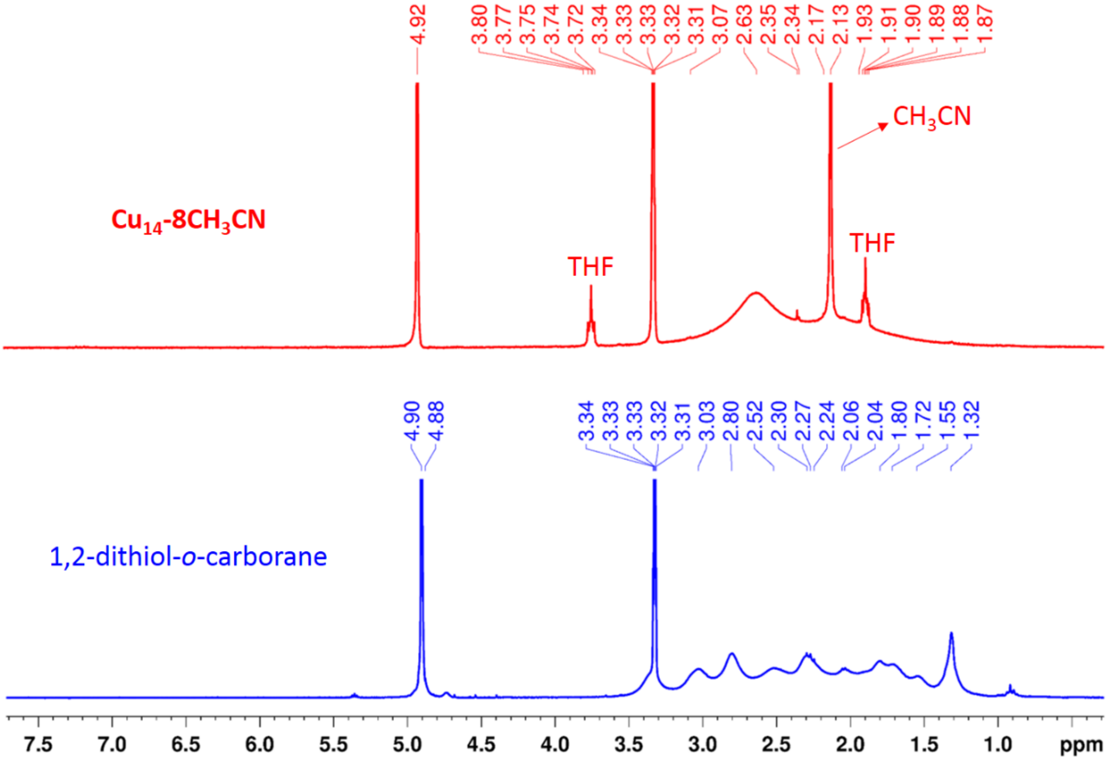

Figure S5. Comparison between ^{1}H NMR (CD_3OD) spectra of **Cu_{14}-8CH_3CN** and 1,2-dithiol-*o*-carborane ligand. The signal for B-H is weak and unstructured due to the limit solubility of **Cu_{14}-8CH_3CN**. Reliable integration was not performed because of the peak overlap.

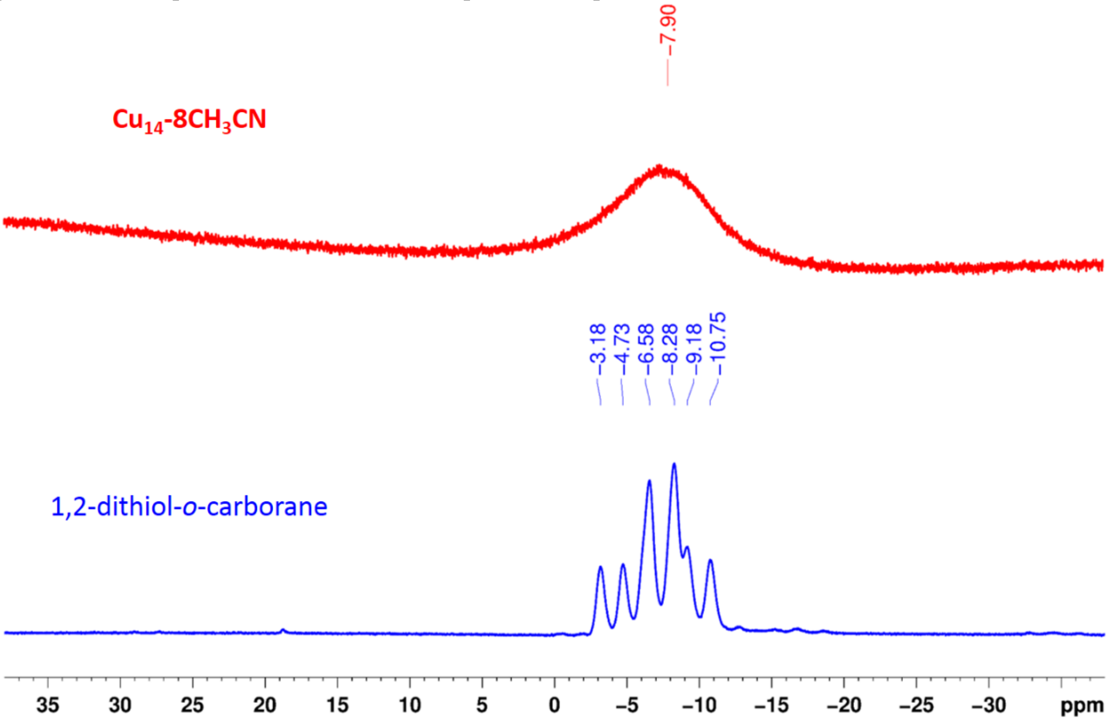

Figure S6. Comparison between ^{11}B NMR spectra of **Cu_{14}-8CH_3CN** and 1,2-dithiol-*o*-carborane ligand. The spectrum of **Cu_{14}-8CH_3CN** shows an unstructured band due to low solubility. The range of the chemical shifts fully coincides in both spectra, indicating that the *closo* structure of carborane is retained in the final **Cu_{14}-8CH_3CN** clusters.

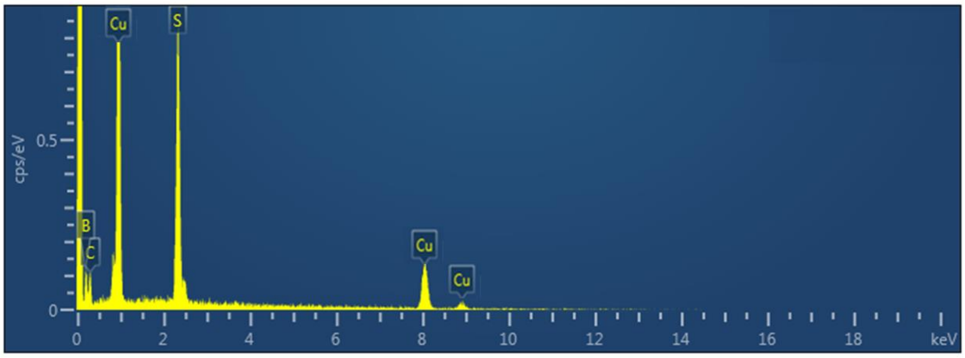

Figure S7. Energy dispersive spectrometer (EDS) mapping of **Cu_{14}-8CH_3CN** crystals.

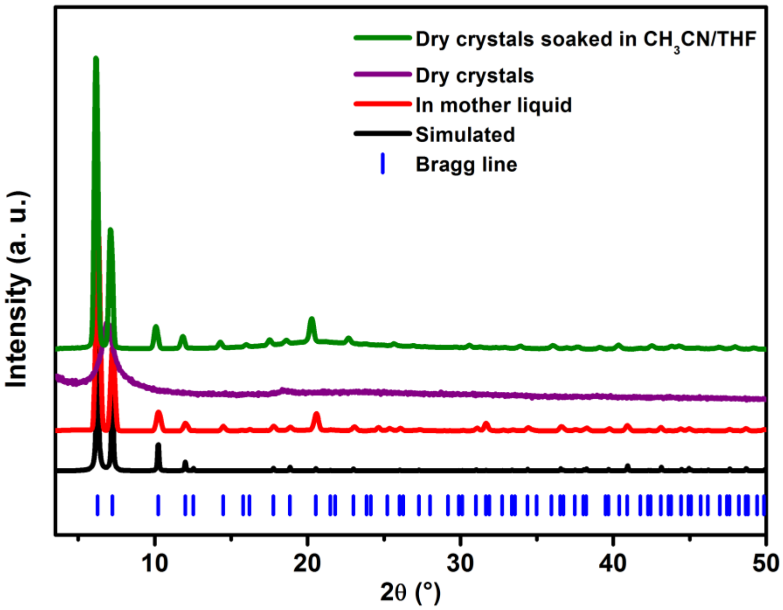

Figure S8. PXRD patterns of **Cu_{14}-8CH_3CN**.

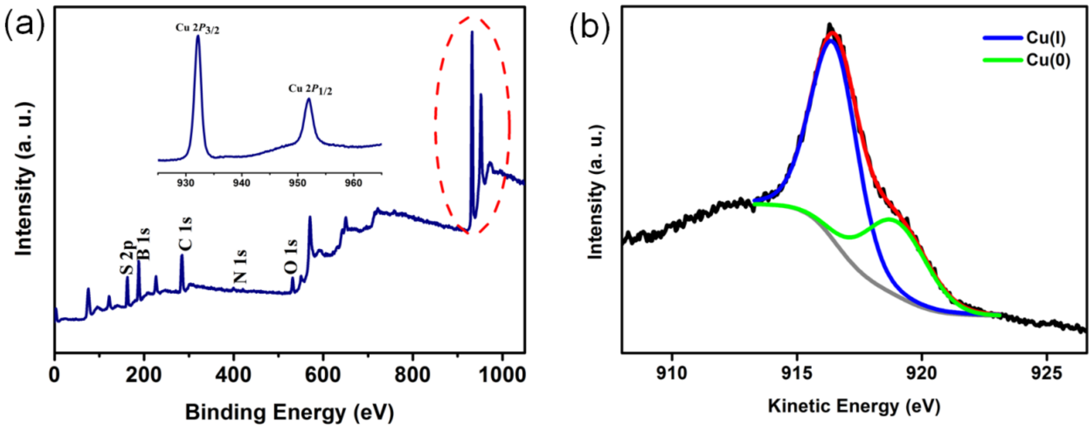

Figure S9. (a) XPS survey spectrum of **Cu_{14}-8CH_3CN**, confirming the presence of Cu, S, C, N, B, and O. The inset shows the expanded XPS spectrum in the Cu 2p region of **Cu_{14}-8CH_3CN**. (b) Cu LMM XAES spectrum of **Cu_{14}-8CH_3CN**.

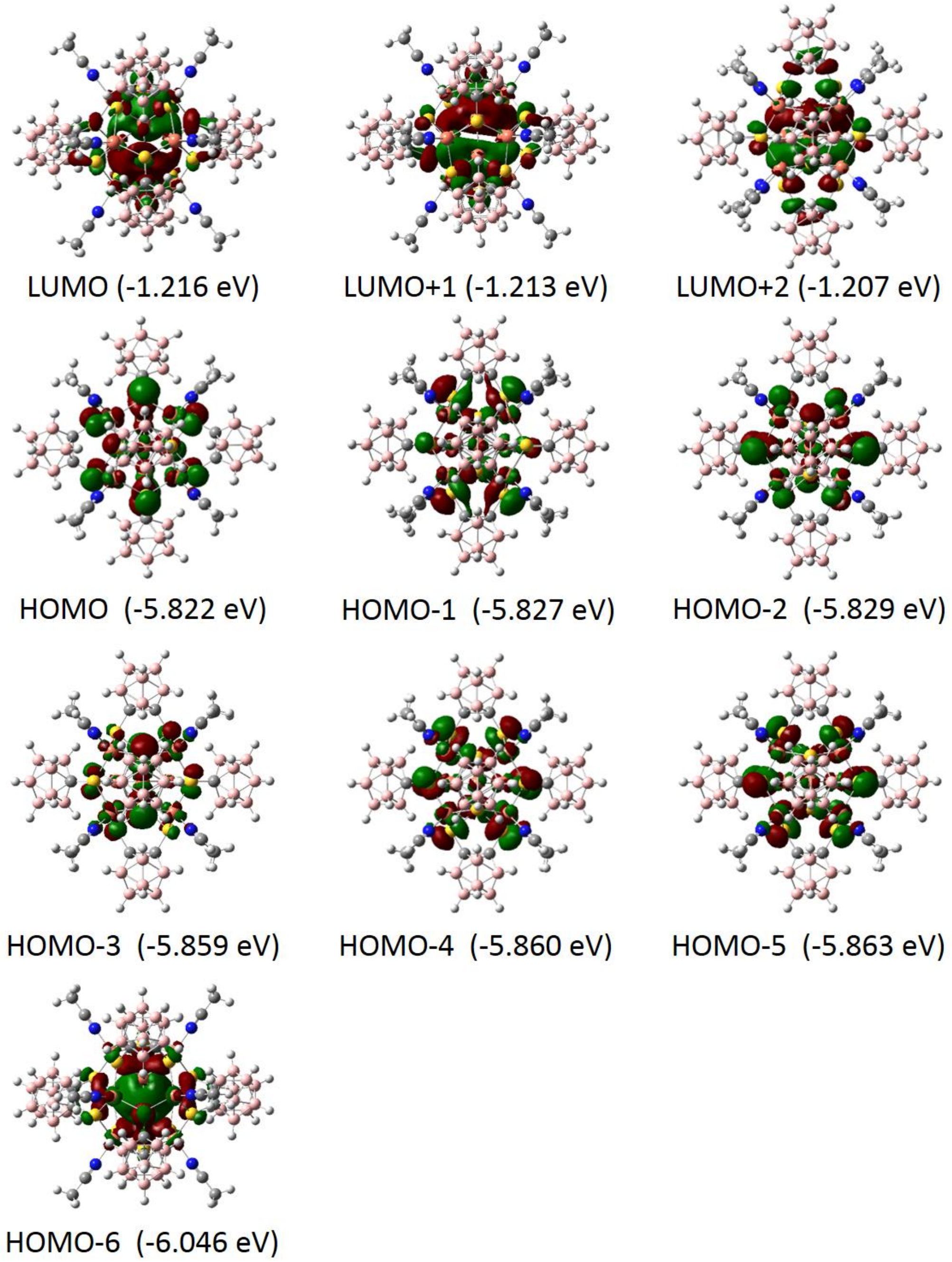

Figure S10. Selected frontier MO representations for the **Cu_{14}-8CH_3CN** cluster. The degenerate lowest unoccupied molecular orbitals (LUMO to LUMO+2) indicate strong superatomic P character over the entire Cu_{14} cube, while the HOMO-6 state shows superatomic S character over the kernel of the cluster.

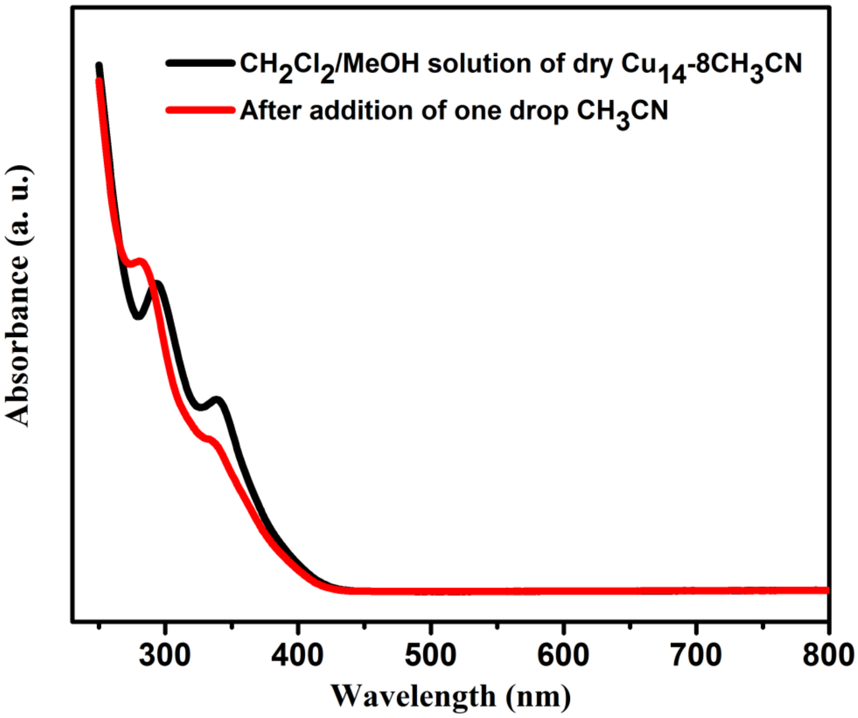

Figure S11. UV-vis absorption spectra of fresh CH_2Cl_2-MeOH solution of dry **Cu_{14}-8CH_3CN** (black) and the solution after the addition of one drop of CH_3CN.

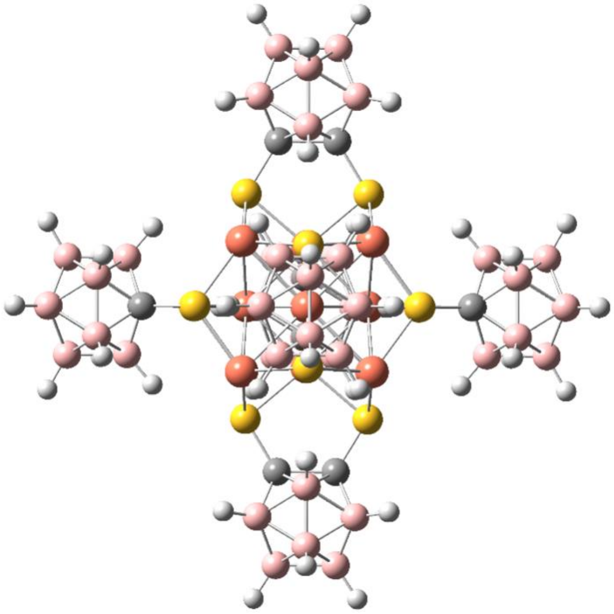

Figure S12. The optimized **Cu_{14}** model. Color codes: orange = copper; yellow = sulfur; gray = carbon; light gray = hydrogen; rose = boron.

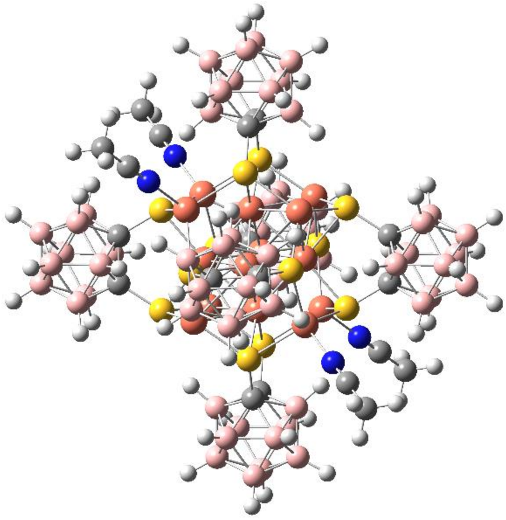

Figure S13. The optimized **Cu_{14}-4CH_3CN** model. Color codes: orange = copper; yellow = sulfur; gray = carbon; light gray = hydrogen; blue = nitrogen; rose = boron.

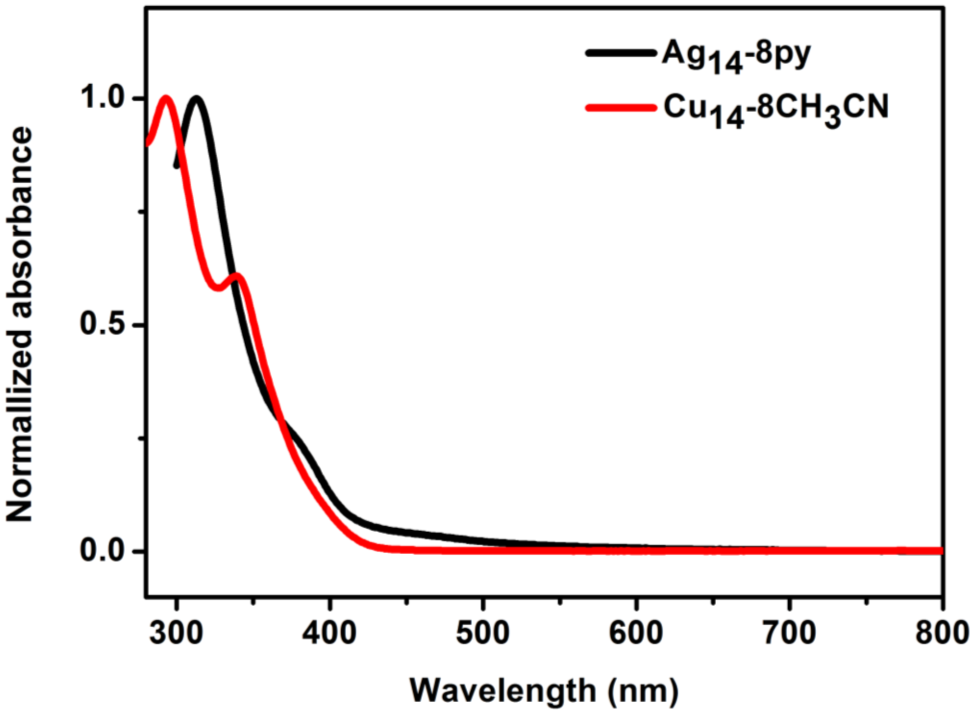

Figure S14. A comparison of the absorption spectra of **Cu_{14}-8CH_3CN** and its Ag analog **Ag_{14}-8py** (py = pyridine).

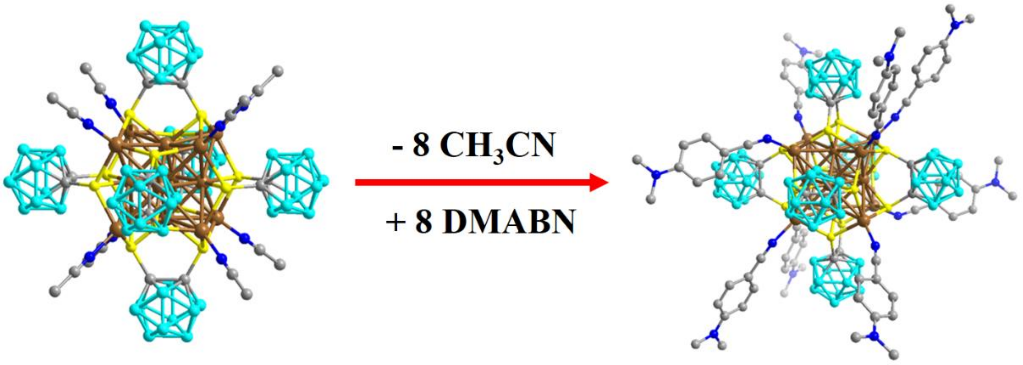

Figure S15. Schematic representation of site-specific substitution of CH_3CN by DMABN. Color codes: brown = copper; yellow = sulfur; gray = carbon; blue = nitrogen; turquoise = boron.

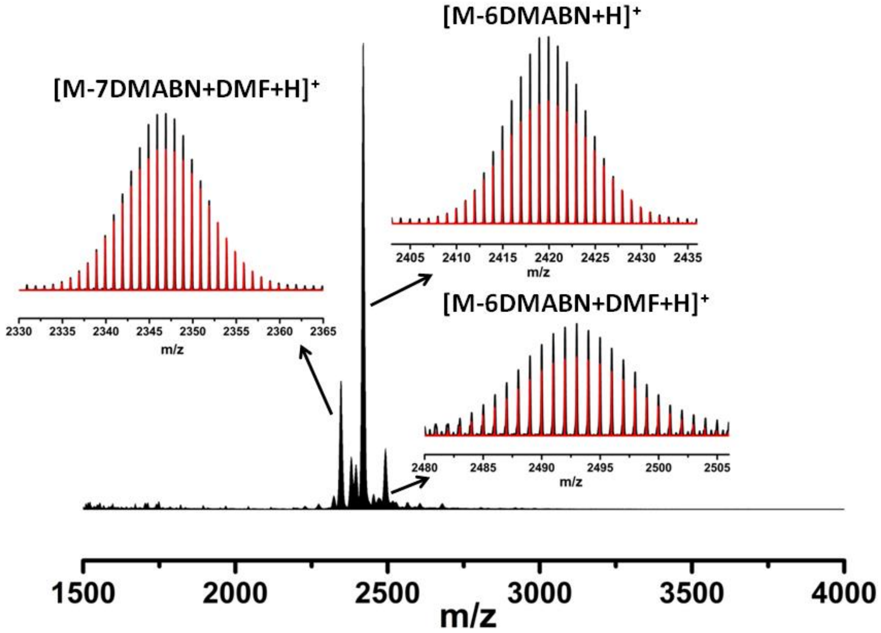

Figure S16. Positive mode ESI-TOF-MS spectrum of **Cu_{14}-8DMABN** in DMF. Inset: Enlarged portion of the spectrum showing the measured (black) and simulated (red) isotopic distribution patterns. m/z for [M-7DMABN+DMF+H]$^+$: calcd 2346.8772, Found 2346.8938; [M-6DMABN+H]$^+$: calcd 2419.9089, Found 2419.9487; [M-6DMABN+DMF+H]$^+$: calcd 2492.9621, Found 2492.9921. (M = $Cu_{14}(C_2B_{10}H_{10}S_2)_6$(DMABN)$_8$).

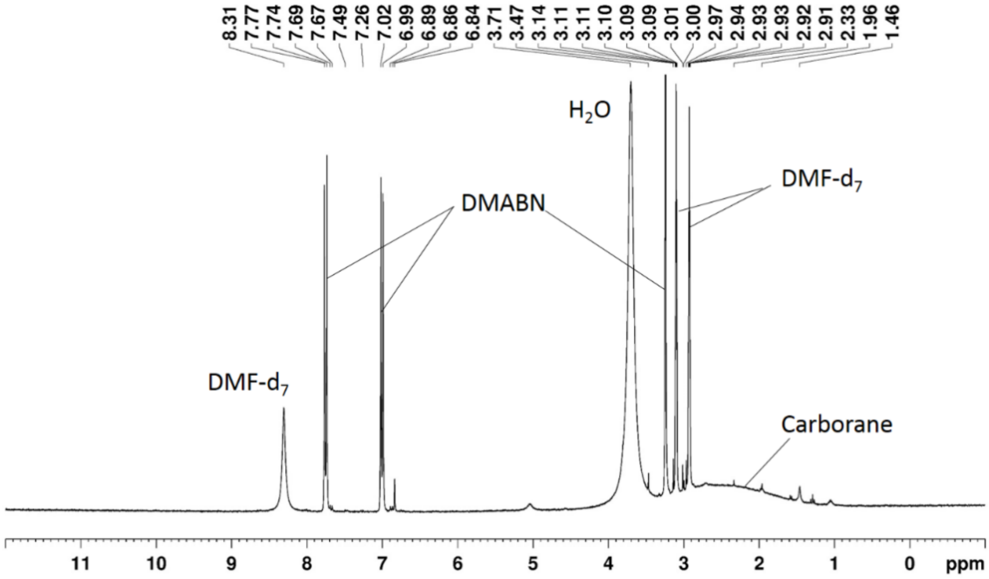

Figure S17. ^{1}H NMR (DMF-d_7) spectrum of **Cu_{14}-8DMABN**.

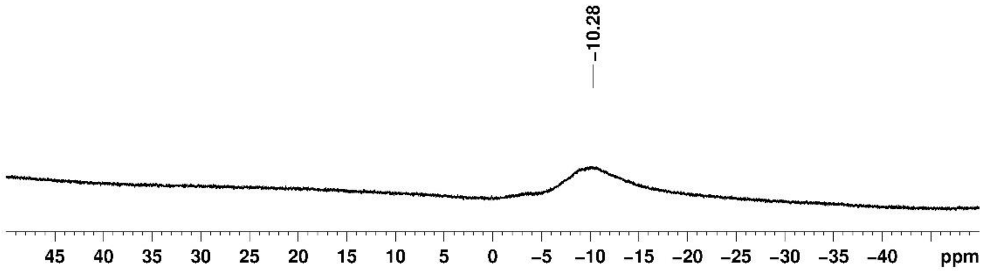

Figure S18. ^{11}B NMR spectrum of **Cu_{14}-8DMABN**.

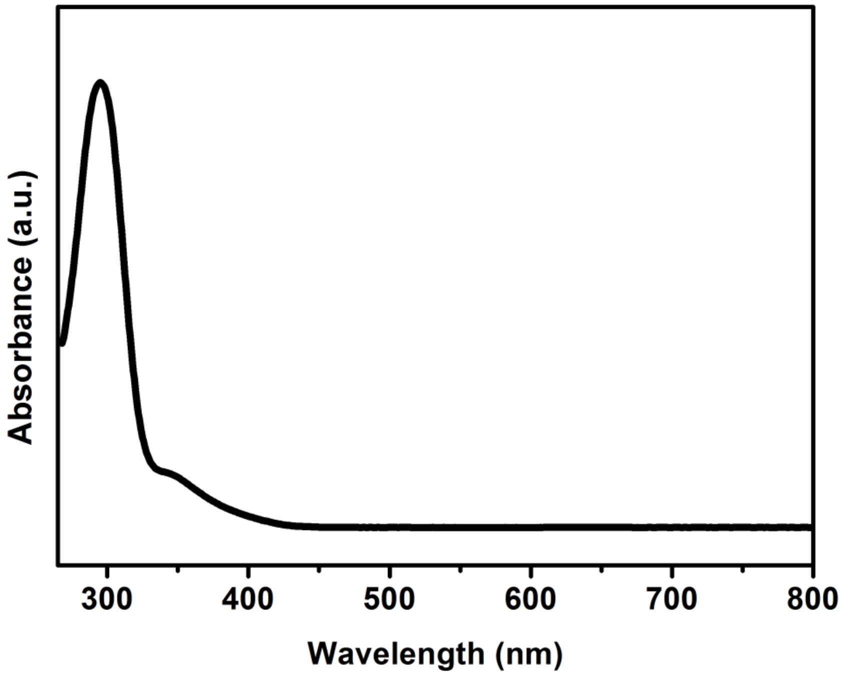

Figure S19. UV-vis spectrum of **Cu_{14}-8DMABN** in DMF.

Electrocatalysis tests.

Ethanol electrooxidation reactions (EERs) evaluation. The EER activities of **Cu_{14}-8CH_3CN** were evaluated in a three-electrode cell with N_2-saturated 0.1 M KOH solutions at room temperature. A glassy carbon (GC) electrode (5 mm in diameter, S = 0.1962 cm^2) was coated with **Cu_{14}-8CH_3CN** as follows: the **Cu_{14}-8CH_3CN** clusters (1.25 mg) and super P (1 mg) was ultrasonicated in a mixture of methanol (100 µL), dichloromethane (100 µL) and Nafion (30 µL) until a uniform ink was achieved. Then, 10 µL of the competent precatalyst ink was pipetted onto the GC electrode surface by using a micropipettor and dried at ambient temperature. After the solvent in 10 µL of slurry was completely evaporated, the known amount of solid sample was left on the surface of GCE to form a uniform precatalyst film. The **Cu_{14}-8CH_3CN** loading amount of GCE was 0.27 mg/cm^2 for ethanol electrocatalytic oxidation. This modified precatalyst film with Nafion cross-linking matrix on the GCE is very stable under the electrochemical test in electrolyte. The GC electrode coated with **Cu_{14}-8CH_3CN** was used as the working electrode, and an Ag/AgCl (KCl saturated) electrode and a platinum wire were used as the reference and counter electrode, respectively. Cyclic voltammograms of the electrocatalysts were detected at a scan rate of 50 mV s^{-1}.

Electrochemical Detection of Hydrogen Peroxide. All measurements were carried out on a CHI 660E electrochemical workstation in a standard three-electrode cell at room temperature. An Ag/AgCl (KCl, saturated) electrode and Pt wire were used as the reference and counter electrode, respectively. A glassy carbon electrode coated with **Cu_{14}-8CH_3CN** cluster solution was used as the working electrode. For the electrochemical detection, a glassy carbon (GC) electrode was first polished with alumina slurries (0.05 µm) and then cleaned by successive sonication in dilute nitric acid solution, ultrapure water, and ethanol respectively. The catalyst solution was prepared by dispersing 0.5 mg **Cu_{14}-8CH_3CN** in 500 µL solution containing 250 µL of methanol, 230 µL of dichloromethane and 20 µL of 5 wt.% Nafion solution followed by ultrasonication for 5 min. 10 µL of catalyst solution was then dropcast onto the clean GC surface by using a micropipettor and the particle film dried at ambient temperature. The **Cu_{14}-8CH_3CN** loading amount of GCE was 0.14 mg/cm^2 for H_2O_2 detection. This modified catalyst film with Nafion cross-linking matrix on the GCE is very stable under the electrochemical test in electrolyte.

ICP-MS (Inductively coupled plasma mass spectrometry) measurements were measured to detect the loss of Cu in electrolyte after the electrocatalytic test. As a result, no Cu component was detected in the electrolyte for ethanol electrooxidation reaction, and a negligible amount of Cu (0.05 ppm) appeared in the electrolyte for H_2O_2 detection, indicating the fastness of catalyst on electrodes.

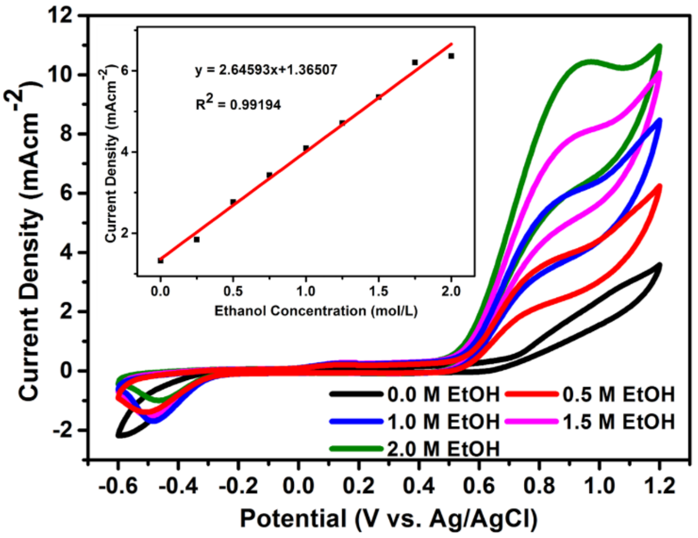

Figure S20. Cyclic voltammograms for **Cu_{14}-8CH_3CN** in 0.1 M KOH solution in the presence of different concentrations of ethanol (0.0, 0.5, 1.0, 1.5 and 2.0 M) (vs. Ag/AgCl, scan rate: 50 mV s^{-1}). Inset: the corresponding relationship between the oxidation peak current density and the concentrations of ethanol.

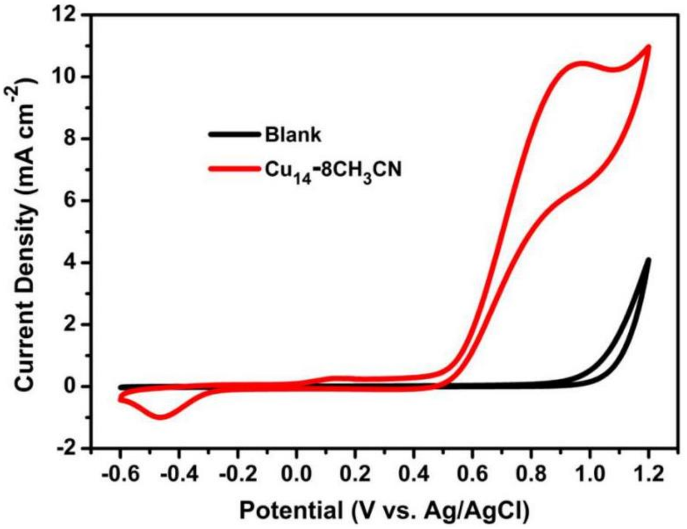

Figure S21. Cyclic voltammograms for **Cu_{14}-8CH_3CN** (red) and blank (black) electrode in 0.1 M KOH solution in the presence of 2.0 M ethanol, respectively. The oxidation current of blank electrode appeared at 1.0-1.2 V (vs. Ag/AgCl), the onset potential of ethanol oxidation for **Cu_{14}-8CH_3CN** was shifted negatively by 500 mV and the corresponding oxidation current was greatly enhanced.

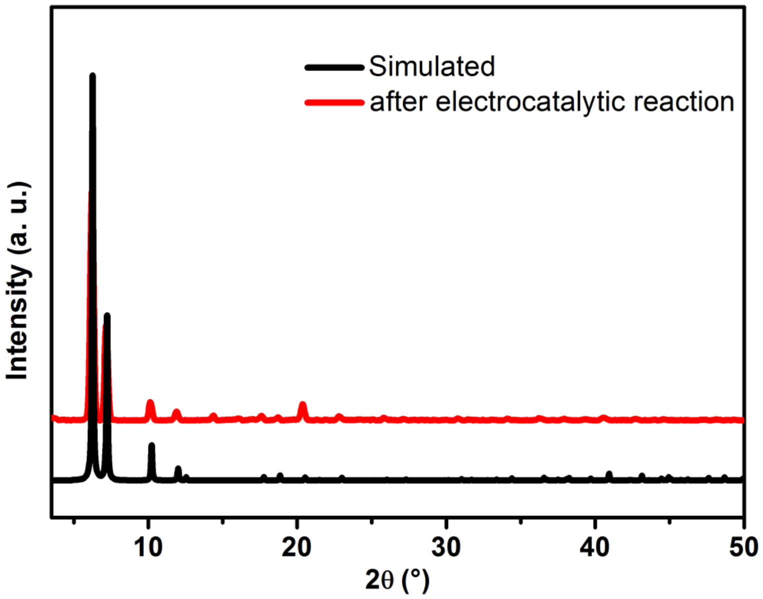

Figure S22. PXRD pattern of the **Cu_{14}-8CH_3CN** sample immersed in CH_3CN/THF after electrocatalytic reaction.

Table S1. Crystallographic data and structure refinement of **Cu_{14}-8CH_3CN**, **Cu_{14}-8DMABN** and **Cu-Disulfide**.

	**Cu_{14}-8CH_3CN**	**Cu_{14}-8DMABN**	**Cu-Disulfide**
CCDC number	1861371	1861372	1886055
Empirical formula	$C_{28}H_{84}Cu_{14}B_{60}N_8S_{12}$	$C_{94}H_{160}Cu_{14}B_{60}O_{2.5}N_{16}S_{12}$	$C_{11}H_{23}B_{9.5}CuN_{4.5}S_2$
Formula weight	2456.06	3477.25	448.69
Temperature / K	200.00(10)	150.00(10)	293(2)
Crystal system	cubic	triclinic	monoclinic
Space group	*Fm*-3*m*	*P*-1	*I*2/*a*
a /Å	24.4525(2)	19.1741(5)	22.0409(9)
b /Å	24.4525(2)	19.9770(5)	8.4392(2)
c /Å	24.4525(2)	21.3399(5)	26.1078(8)
α /°	90	85.459(2)	90
β /°	90	82.316(2)	106.511(4)
γ /°	90	89.023(2)	90
Volume /$Å^3$	14620.7(3)	8075.1(4)	4656.0(3)
Z	4	2	8
ρ_{calc} g/cm^3	1.116	1.430	1.280
μ /mm^{-1}	3.903	3.735	3.022
F(000)	4824.0	3508.0	1832.0
Crystal size/mm^3	0.14 × 0.06 × 0.04	0.28 × 0.26 × 0.25	0.20 × 0.20 × 0.05
Radiation	Cu Kα (λ = 1.54184)	Cu Kα (λ = 1.54184)	Cu Kα (λ = 1.54184)
2Θ range for data collection /°	6.26 to 134.562	4.65 to 132	7.06 to 135
Index ranges	$-16 \leq h \leq 18$, $-26 \leq k \leq 19$, $-29 \leq l \leq 0$	$-22 \leq h \leq 22$, $-23 \leq k \leq 23$, $-25 \leq l \leq 18$	$-24 \leq h \leq 26$, $-9 \leq k \leq 8$, $-23 \leq l \leq 31$
Reflections collected	3273	84169	10586
Independent reflections	710 [R_{int} = 0.0209, R_{sigma} = 0.0229]	27995 [R_{int} = 0.0643, R_{sigma} = 0.0683]	4171 [R_{int} = 0.0602, R_{sigma} = 0.0653]
Data/restraints/parameters	710/7/55	27995/290/1871	4171/9/268
Goodness-of-fit on F^2	1.182	1.052	1.025
Final *R* indexes [I>=2σ (I)]	R_1 = 0.0400, wR_2 = 0.1459	R_1 = 0.0996, wR_2 = 0.2745	R_1 = 0.0586, wR_2 = 0.1577
Final *R* indexes [all data]	R_1 = 0.0448, wR_2 = 0.1551	R_1 = 0.1419, wR_2 = 0.3050	R_1 = 0.0677, wR_2 = 0.1649
Largest diff. peak/hole / e $Å^{-3}$	0.34/–0.96	1.53/–0.62	0.54/-0.43

$R_1 = \sum||F_o|-|F_c||/\sum|F_o|$. $wR_2 = [\sum w(F_o^2 - F_c^2)^2/\sum w(F_o^2)^2]^{1/2}$

Table S2. Transition energy, oscillator strength, and orbital contributions of the strongest electronic excitations of **Cu_{14}-4CH_3CN**.

Wavelength (nm)	Osc. Strength	Major orbital contributions (only those ⩾15% are shown here)
350.473	0.0319	H-3->LUMO (52%), HOMO->L+1 (28%)
346.1095	0.0347	H-5->LUMO (81%)
340.0903	0.0522	H-5->L+1 (53%), H-3->L+1 (23%)
331.9945	0.0724	H-5->L+2 (40%), H-3->L+2 (25%)
325.1596	0.1615	H-6->L+1 (52%), H-5->L+2 (27%)
324.7763	0.0349	H-9->L+1 (17%), H-7->LUMO (55%)
317.5233	0.0831	H-12->LUMO (47%), H-6->L+2 (22%)
316.5748	0.0433	H-7->L+1 (78%)
311.1953	0.0419	H-12->L+1 (15%), H-9->L+2 (61%)
306.9046	0.0359	H-12->L+2 (80%)
297.865	0.0386	H-17->LUMO (20%), H-15->LUMO (61%)
295.91	0.0856	H-17->LUMO (45%), H-14->LUMO (17%)
294.8194	0.0534	H-14->LUMO (66%)
289.3157	0.081	H-17->L+1 (37%), H-15->L+1 (33%)
288.434	0.0716	H-16->L+1 (19%), H-15->L+2 (24%), H-14->L+2 (39%)
286.6733	0.0463	H-16->L+2 (40%)
286.66	0.1051	H-16->L+1 (22%), H-16->L+2 (16%), H-15->L+2 (30%)
285.4194	0.1052	H-20->LUMO (61%), H-15->L+1 (18%)
282.0239	0.0513	H-17->L+2 (78%)
274.0205	0.0316	H-20->L+2 (27%), H-1->L+3 (29%)
272.401	0.0746	H-2->L+3 (55%)
270.6469	0.0601	H-2->L+4 (47%)
270.1162	0.0615	H-1->L+3 (24%), H-1->L+4 (53%)

3. References

[1] C. Viñas, R. Benakki, F. Teixidor, J. Casabó, *Inorg. Chem.* **1995**, *34*, 3844-3845.

[2] CrysAlisPro 2012, Agilent Technologies. Version 1.171.36.31.

[3] G. M. Sheldrick, *Acta Cryst. A* **2015**, *71*, 3-8.

[4] G. M. Sheldrick, *Acta Cryst. A* **2008**, *64*, 112-122.

[5] O. V. Dolomanov, L. J. Bourhis, R. J. Gildea, J. A. K. Howard, H. Puschmann, *J. Appl. Cryst.* **2009**, *42*, 339-341.

[6] K. Brandenburg, *Diamond*, **2010**.

[7] M. J. Frisch, G. W. Trucks, H. B. Schlegel, G. E. Scuseria, M. A. Robb, J. R. Cheeseman, G. Scalmani, V. Barone, G. A. Petersson, H. Nakatsuji, X. Li, M. Caricato, A. V. Marenich, J. Bloino, B. G. Janesko, R. Gomperts, B. Mennucci, H. P. Hratchian, J. V. Ortiz, A. F. Izmaylov, J. L. Sonnenberg, D. Williams-Young, F. Ding, F. Lipparini, F. Egidi, J. Goings, B. Peng, A. Petrone, T. Henderson, D Ranasinghe, V. G. Zakrzewski, J. Gao, N. Rega, G. Zheng, W. Liang, M. Hada, M. Ehara, K. Toyota, R. Fukuda, J. Hasegawa, M. Ishida, T. Nakajima, Y. Honda, O. Kitao, H. Nakai, T. Vreven, K. Throssell, Jr. J. A. Montgomery, J. E. Peralta, F. Ogliaro, M. J. Bearpark, J. J. Heyd, E. N. Brothers, K. N. Kudin, V. N. Staroverov, T. A. Keith, R. Kobayashi, J. Normand, K. Raghavachari, A. P. Rendell, J. C. Burant, S. S. Iyengar, J. Tomasi, M. Cossi, J. M. Millam, M. Klene, C. Adamo, R. Cammi, J. W. Ochterski, R. L. Martin, K. Morokuma, O. Farkas, J. B. Foresman, D. J. Fox, *Gaussian, Inc.*, Wallingford CT, **2016**.

[8] J. P. Perdew, K. Burke, M. Ernzerhof, Generalized Gradient Approximation Made Simple. *Phys. Rev. Lett.* **1996**, *77*, 3865-3868.

[9] F. Weigend, R. Ahlrichs, *Phys. Chem. Chem. Phys.* **2005**, *7*, 3297-3305.

[10] N. M. O’Boyle, A. L. Tenderholt, K. M. Langner, *J. Comp. Chem.* **2008**, *29*, 839-845.

[11] T. Lu, F. W. Chen, *J. Comput. Chem.* **2012**, *33*, 580-592.

[12] X. Zhang, Z. Zhou, H. Yan, *Chem. Commun.* **2014**, *50*, 13077-13080.

4. Caption of videos

Video 1. Video showing the self-reduction process (avi)
